# Temporal Measures in Cardiac Structure and Function During the Development of Obesity Induced by Different Types of Western Diet in a Rat Model

**DOI:** 10.3390/nu12010068

**Published:** 2019-12-26

**Authors:** Danielle Fernandes Vileigas, Cecília Lume de Carvalho Marciano, Gustavo Augusto Ferreira Mota, Sérgio Luiz Borges de Souza, Paula Grippa Sant’Ana, Katashi Okoshi, Carlos Roberto Padovani, Antonio Carlos Cicogna

**Affiliations:** 1 Department of Internal Medicine, Botucatu Medical School, São Paulo State University, UNESP, Botucatu 18618687, Brazil; dani.vileigas@gmail.com (D.F.V.); cecilialcm3@gmail.com (C.L.d.C.M.); gamota@alunos.fmb.unesp.br (G.A.F.M.); enfeborges@gmail.com (S.L.B.d.S.); paulagrippa@yahoo.com.br (P.G.S.); katashi.okoshi@unesp.br (K.O.); 2 Department of Biostatistics, Institute of Biosciences, São Paulo State University, Botucatu 18618970, Brazil; cr.padovani@unesp.br

**Keywords:** cardiac remodeling, cardiac dysfunction, echocardiogram, obese rats, high-fat high-sugar diet

## Abstract

Obesity is recognized worldwide as a complex metabolic disorder that has reached epidemic proportions and is often associated with a high incidence of cardiovascular diseases. To study this pathology and evaluate cardiac function, several models of diet-induced obesity (DIO) have been developed. The Western diet (WD) is one of the most widely used models; however, variations in diet composition and time period of the experimental protocol make comparisons challenging. Thus, this study aimed to evaluate the effects of two different types of Western diet on cardiac remodeling in obese rats with sequential analyses during a long-term follow-up. Male Wistar rats were distributed into three groups fed with control diet (CD), Western diet fat (WDF), and Western diet sugar (WDS) for 41 weeks. The animal nutritional profile and cardiac histology were assessed at the 41st week. Cardiac structure and function were evaluated by echocardiogram at four different moments: 17, 25, 33, and 41 weeks. A noninvasive method was performed to assess systolic blood pressure at the 33rd and 41st week. The animals fed with WD (WDF and WDS) developed pronounced obesity with an average increase of 86.5% in adiposity index at the end of the experiment. WDF and WDS groups also presented hypertension. The echocardiographic data showed no structural differences among the three groups, but WDF animals presented decreased endocardial fractional shortening and ejection fraction at the 33rd and 41st week, suggesting altered systolic function. Moreover, WDF and WFS animals did not present hypertrophy and interstitial collagen accumulation in the left ventricle. In conclusion, both WD were effective in triggering severe obesity in rats; however, only the WDF induced mild cardiac dysfunction after long-term diet exposure. Further studies are needed to search for an appropriate DIO model with relevant cardiac remodeling.

## 1. Introduction

Obesity is a complex metabolic disorder recognized worldwide as a significant health concern, and its prevalence has reached epidemic proportions [1]. In 2016, more than 1.9 billion adults were overweight; of these, over 650 million were obese [2]. The etiology of obesity is complex and multifactorial, especially involving environmental and genetic factors. However, the modern obesity epidemic is undoubtedly the result of environmental determinants and is often associated with a reduction in physical activity and increased intake of diets high in saturated fat and sugars, commonly termed Western diet [3,4]. Excessive body fat is the leading risk factor for numerous comorbidities, most notably gastrointestinal diseases, type 2 diabetes mellitus, certain types of cancer, and cardiovascular disease (CVD) [5].

A number of clinical and animal studies have generated convincing evidence that hemodynamic, neurohormonal, and metabolic alterations, which are commonly found in obesity, contribute to changes in cardiac morphology that may predispose to impaired ventricular function and heart failure [6,7]. For a better understanding of the mechanisms underlying cardiac dysfunction in obesity and to assess potential treatments for this pathology, several experimental animal models have been developed, and one of the most commonly used is the diet-induced obesity (DIO) model [8]. Among the various types of experimental diets, such as high-fat, cafeteria, and high-fructose, many studies have preferably used the Western diet (WD) due to its relative similarity to the human consumption responsible for the obesity epidemic [9,10].

In WD, various sources of fat and sugar are used in different proportions, sometimes higher in fat or higher in sugar, and there is no consensus on which one is the most effective to cause cardiac dysfunction; moreover, the duration of experimental studies ranges widely [11,12,13,14,15,16]. Thus, a considerable divergence of results concerning heart function is observed among different studies making comparisons challenging. Despite the existence of these several investigations regarding Western diet-induced cardiac dysfunction in animal models, a direct comparison between different patterns of WD on cardiac structure and function over time has not been adequately evaluated so far. Therefore, this study aimed to assess the effects of two types of the Western diet, one with high fat (50% fat, 35% carbohydrate) and one with high carbohydrate (34% fat, 49% carbohydrate) content, on cardiac remodeling in obese rats with sequential analyses during a long-term follow-up. This proposal may be useful to determine an adequate obesity model with functional impairment of the heart so that pathophysiological mechanisms or preventive and therapeutic strategies can be investigated in future studies.

## 2. Material and Methods

### 2.1. Animals

Male *Wistar* rats (60 days old) were obtained from our breeding colony and housed in individual cages under a controlled environment with 12 h light/dark cycle at room temperature (24 °C ± 2 °C) and 55 ± 5% humidity with water and food ad libitum. All animal experiments and procedures were performed according to the Guide for the Care and Use of Laboratory Animals published by U.S. National Institutes of Health [17] and were approved by the Ethics Committee on Animal Experiments of the Botucatu Medical School, São Paulo State University, UNESP (protocol 1119/2015-CEUA).

### 2.2. Experimental Design

**Figure 1:**
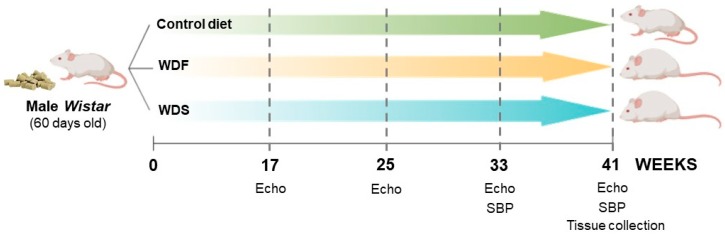
Schematic representation of the experimental design for the study of the effects of dietary interventions using a rat model. WDF and WDS: Western diet fat and sugar, respectively. Echo: echocardiographic analysis. SBP: systolic blood pressure.

The experimental timeline for this study was conducted as shown in Figure 1. Randomized rats were fed with a control diet (CD), Western diet fat (WDF), or Western diet sugar (WDS) for 41 weeks (*n* = 10 for each group). The echocardiographic analysis was performed 17 weeks after the beginning of the study and repeated every 2 months until completion at 41 weeks.

After 41 weeks of experimental protocol, following the echocardiogram and systolic blood pressure evaluation, the animals were fasted overnight (12 h), anesthetized (50 mg/kg ketamine; 10 mg/kg xylazine; intraperitoneal injection), and sacrificed by decapitation. The heart was rapidly isolated; perfused with phosphate-buffered saline (PBS) to remove blood; and then the left and right ventricles, atria, and papillary muscle were dissected for further analysis. White adipose tissues (WATs) were also isolated, dissected, and weighed for nutritional profile assessment.

### 2.3. Diet Composition

**Figure 2:**
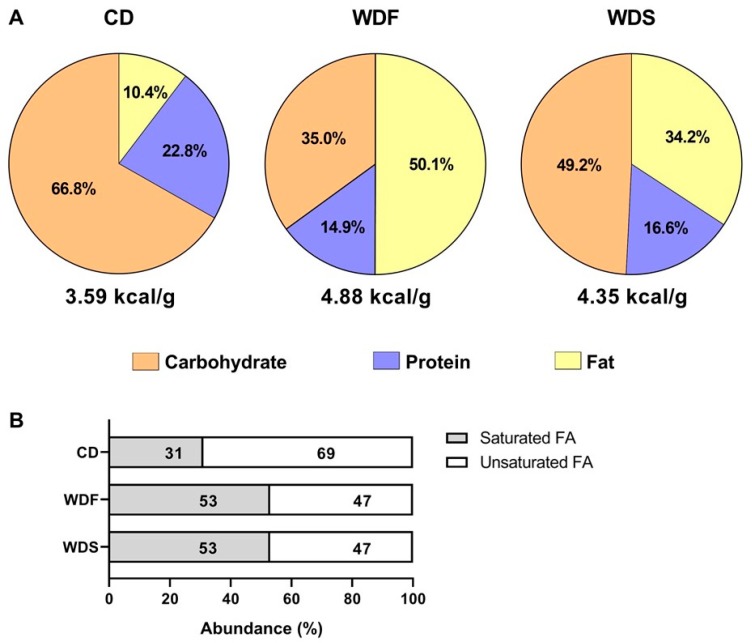
Nutritional composition of diets used in the study. (**A**) Percentage of total calories from carbohydrate (orange), protein (blue), and fat (yellow) in the Control diet (CD), Western diet fat (WDF), and Western diet sugar (WDS). (**B**) Relative abundance of saturated and unsaturated fatty acids (FA) in all diets.

Diets were developed at the Experimental Research Unit (UNIPEX-UNESP) using the following ingredients: soybean meal, soybean hull, corn bran, dextrin, sucrose, fructose, soybean oil, palm oil, palm kernel oil, lard, salt, and vitamin and mineral premix. The CD was custom-formulated with the same ingredients as the WDF and WDS except for fructose, sucrose, palm oil, and lard added only in the WDF and WDS, and soybean oil added only in the CD to produce three different diets in fat, protein, and carbohydrate contents (Table 1 and Figure 2). The CD and WDF have been used in our previous study [18].

### 2.4. Nutritional Profile of the Animals

The nutritional profile was assessed according to the following parameters: energy intake, feed efficiency, body weight, white adipose tissues (WATs) weight, and adiposity index (AI).

Calorie consumption was determined by multiplying the energy value of each diet by the food intake (g × kcal). The feed efficiency was calculated to analyze the animal’s capacity to convert consumed food energy in body weight, dividing the total body weight gain (g) by total energy intake (kcal). The animals were weighed once a week. The WATs were determined by the sum of epididymal, retroperitoneal, and visceral fat pad weights. To calculate the AI, the WATs was divided by the final body weight as follows: (WATs/final body weight) × 100. This index is an easy and consistent method to evaluate the amount of body fat in rodents and several authors have used it [9,19,20].

### 2.5. Systolic Blood Pressure Evaluation

Systolic blood pressure (SBP) was measured in conscious rats at the 33rd and 41st week using the noninvasive tail-cuff method with an electro-sphygmomanometer, Narco Bio-System (International Biomedical, Austin, TX, USA), as previously described [21]. The rats were warmed in a wooden box between 38 and 40 °C with heat generated by two incandescent lamps for 4 min to cause vasodilation of tail artery. Then, they were transferred to a cylindrical iron support that was specially designed to allow total exposure of the animal’s tail. A sensor coupled to the electro-sphygmomanometer was placed in the proximal region of the tail. The arterial pulsations were recorded in a computerized data acquisition system (AcqKnowledge ^®^ MP100, Biopac Systems Inc., Santa Barbara, CA, USA). The average of two readings was recorded for each measurement.

### 2.6. Echocardiographic Study

The first echocardiographic analysis was performed at the 17th week of dietary treatment and evaluated every 2 months until the 41st week, totaling four moments of study (17, 25, 33, and 41 weeks). The analysis was performed using commercially available echocardiography (General Electric Medical Systems, Vivid S6, Tirat Carmel, Israel) equipped with a 5–11.5 MHz multi-frequency transducer, as previously described [22]. Rats were anesthetized by intraperitoneal injection of a mixture of ketamine (50 mg/kg) and xylazine (1 mg/kg). Two-dimensionally guided M-mode images were obtained from short-axis views of the LV at or just below the tip of the mitral valve leaflets and at the level of the aortic valve and left atrium. Flow evaluation (E and A waves) and tissue Doppler were performed in the apical four-chamber view. The parameters used to calculate the Tei index were obtained in the apical five-chamber view. M-mode images of the LV were printed on a black and white thermal printer (Sony UP-890MD) at a sweep speed of 100 mm/sec. The same observer manually measured all LV structures according to the method of the American Society of Echocardiography [23]. The measurements obtained were the mean of at least five cardiac cycles on the M-mode tracings.

The following LV structural parameters were analyzed: LV diastolic diameter (LVDD), LV diastolic posterior wall thickness (DPWT), LV relative wall thickness (RWT), and diameters of the left atrium (LA) and aorta (AO). LV function was assessed by the following parameters: endocardial fractional shortening (EFS), ejection fraction (EF) calculated by the Cube method, and early and late diastolic mitral inflow velocities (E and A waves) ratio. A combined diastolic and systolic LV function was measured, calculating the myocardial performance index (Tei index). The study was complemented using tissue Doppler imaging (TDI) to evaluate early diastolic (E’) velocity of the mitral annulus (arithmetic average of the lateral and septal walls) and the ratio E/E’.

### 2.7. Cardiac Morphological Profile

The following parameters determined macroscopic cardiac remodeling: heart, atria, and left and right ventricle weights, as well as their ratio with tibia length. Additionally, frozen LV samples were used for histological analysis, as previously described [22]. LV transverse sections were cut at 5 µm thickness in a cryostat cooled to −20 °C and then stained with hematoxylin and eosin to determine transverse myocyte diameter, which was measured in at least 50–70 myocytes from each LV as the shortest distance between borders drawn across the nucleus. Collagen interstitial fraction was also determined using picrosirius red staining of LV sections and, on average, 20 microscopic fields were used to quantify interstitial collagen fractional area. Perivascular collagen was excluded from this analysis. All measurements were performed using a Leica microscope (magnification 40×) attached to a video camera and connected to a computer equipped with image analysis software (Image-Pro Plus 3.0, Media Cybernetics, Silver Spring, MD, USA).

### 2.8. Statistical Analysis

All data were tested for normality before statistical analysis using the Shapiro-Wilk test. The results were analyzed using One-way ANOVA followed by Tukey post hoc test (parametric distribution of data) or Kruskal-Wallis test followed by Dunn’s (non-parametric distribution). Two-way ANOVA with repeated measures followed by Bonferroni post hoc test was used to determine statistical differences in body weight evolution, SBP, and echocardiogram. Data are expressed as mean ± s.e.m. (standard error of the mean) or median (maximum [Max] and minimum [Min] values). All statistical analyses were performed using SigmaPlot 12.0 (Systat Software, Inc., San Jose, CA, USA), and graphics were generated using GraphPad Prism 8 (GraphPad Software Inc., San Diego, CA, USA). The differences were considered statistically significant when *p* < 0.05.

## 3. Results

### 3.1. Nutritional Profile of the Animals

**Figure 3:**
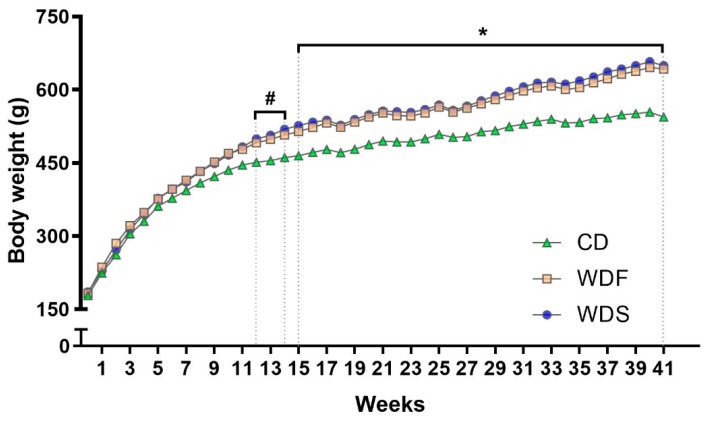
Body weight evolution during dietary intervention with Control diet (CD), Western diet fat (WDF), and Western diet sugar (WDS) for 41 weeks. Data are presented as mean ± s.e.m. Two-way repeated-measures ANOVA and Bonferroni’s multiple comparisons test. ^#^ *p* < 0.05 Control vs. WDS; * *p* < 0.05 Control vs. WDF and WDS (*n* = 10 rats per group).

Over the 41 weeks, both WD induced a progressive increase in body weight gain. The rats fed with WDS became heavier than those fed with CD diet from the 12th week of dietary treatment until the end of the study, while rats fed with WDF showed higher body weight than CD rats only after the 15th week (Figure 3).

As observed in Table 2, the energy intake (kcal per rat) was similar in all groups. After 41 weeks, the rats receiving WDF and WDS showed an average increase of 18% in body weight, 126% in WATs, and 86% in adiposity index, indicating that both diets were equally significant in triggering severe obesity in comparison to CD. Moreover, the feed efficiency was higher in rats fed with WDF and WDS, even though these rats did not consume more calories than rats fed CD, suggesting that weight gain occurred regardless of energy intake.

### 3.2. Systolic Blood Pressure Evaluation

**Figure 4:**
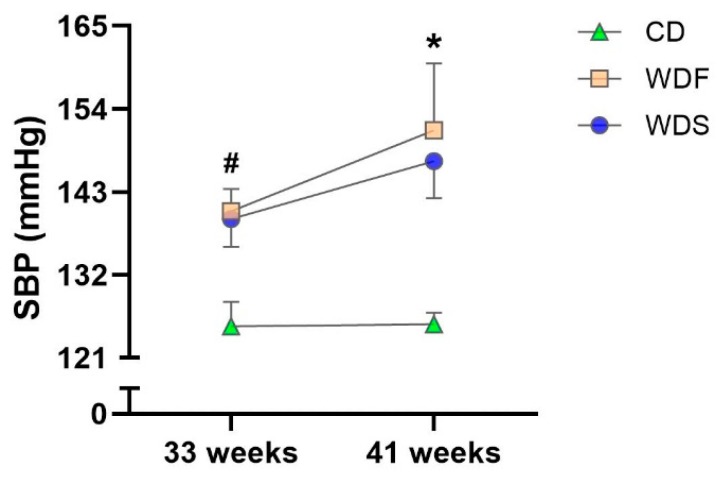
Systolic blood pressure (SBP) at 33rd and 41st week of dietary intervention with Control diet (CD), Western diet fat (WDF), and Western diet sugar (WDS). Data are presented as mean ± s.e.m. Two-way repeated-measures ANOVA and Bonferroni’s multiple comparisons test. * *p* < 0.05 Control vs. WDF and WDS; ^#^ *p* < 0.05 Control vs. WDF (*n* = 7–10 rats per group).

The SBP was higher in WDF-fed animals than their respective controls at 33 and 41 weeks. In the WDS group, there was a trend toward increased SBP (*p* = 0.052) at 33 weeks and significant elevation of the SBP at 41 weeks compared to the CD. These findings indicate that both WD were able to trigger hypertension in the animals (Figure 4).

### 3.3. Cardiac Structural and Functional Assessment

**Figure 5:**
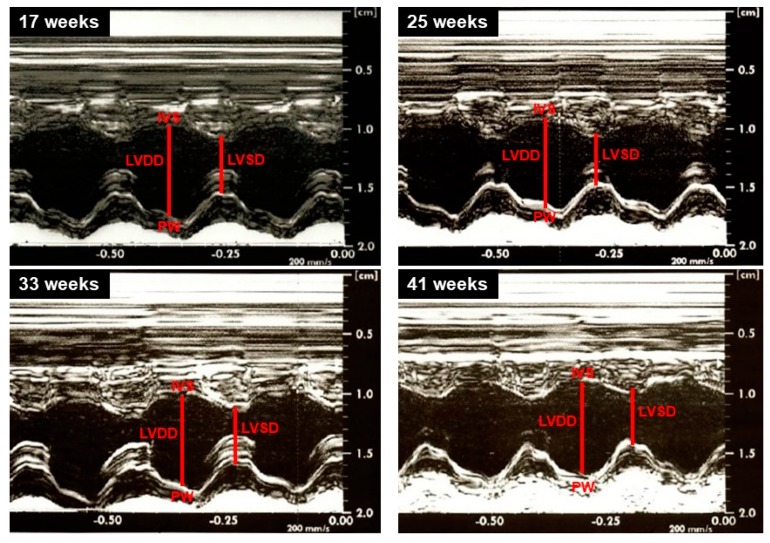
Illustrative left ventricle M-mode echocardiograms from rats at 17th, 25th, 33rd, and 41st week. LVDD and LVSD: left ventricular diastolic and systolic diameters, respectively; PW: left ventricle posterior wall; IVS: interventricular septum.

**Figure 6:**
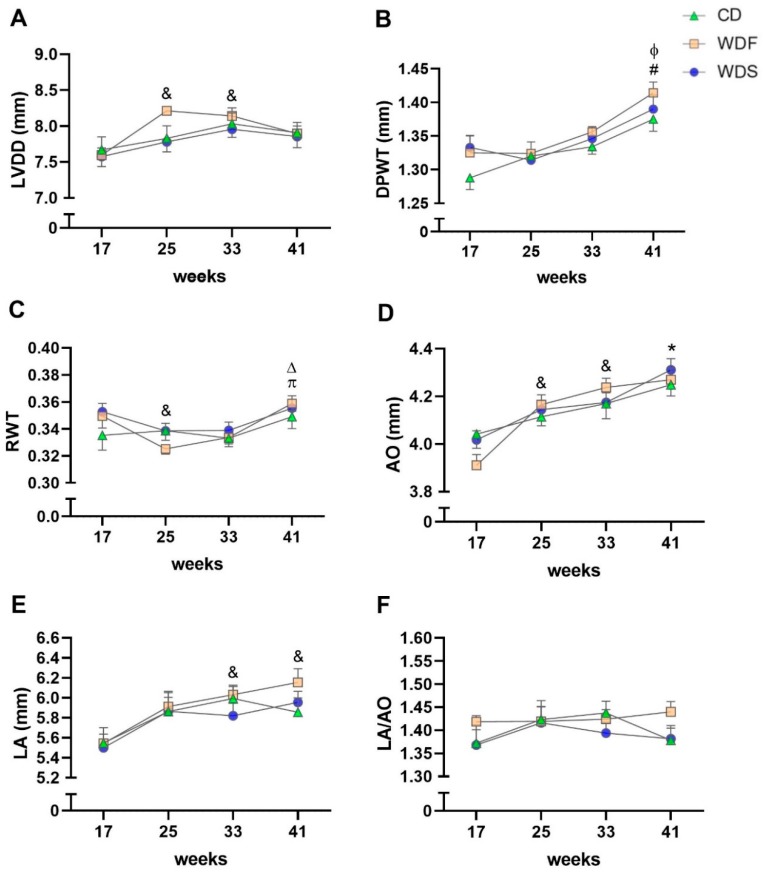
Serial echocardiographic structural assessment. The analysis was performed at the 17th, 25th, 33rd, and 41st week of dietary intervention with Control diet (CD), Western diet fat (WDF), and Western diet sugar (WDS). (**A**) Left ventricle (LV) diastolic diameter (LVDD). (**B**) LV diastolic posterior wall thickness (DPWT). (**C**) Relative wall thickness (RWT). (**D**) Aortic diameter (AO). (**E**) Left atrial diameter (LA). (**F**) LA/AO ratio. Data are presented as mean ± s.e.m. Two-way repeated-measures ANOVA and Bonferroni post hoc test. Symbols indicate differences between the moments fixed the group. **^&^** *p* < 0.05 vs. 17 weeks for WDF; **^Δ^** vs. 33 weeks for WDF; **^ɸ^** *p* < 0.01 vs. 25 weeks for WDF and WDS; * vs. 17 weeks for C, WDF, and WDS; **^#^** *p* < 0.001 vs. 17 weeks for C and WDF; **^π^** vs. 25 weeks for WDF (*n* = 10 per group).

Illustrative LV M-mode echocardiograms are shown in Figure 5. Structural analysis of the heart performed by echocardiogram revealed no significant changes among the CD, WDF, and WDS groups for all variables (Figure 6). Regarding the evaluated moments, LVDD was increased in the WDF group at the 25th and 33rd weeks when compared to the 17th week (Figure 6A). DPWT was increased at 41 weeks compared to 17 weeks in CD and WDF groups; moreover, this variable increased from the 25th week to 41st week in the WDF and WDS groups (Figure 6B). RWT showed a decrease from week 17 to week 25 and augmented from weeks 25 and 33 to week 41 in the WDF group (Figure 6C). AO enlarged at weeks 25 and 33 compared to week 17 in WDF-fed rats; furthermore, this variable increased from the 17th week to the 41st week in all groups (Figure 6D). LA expanded from week 17 to weeks 33 and 41 only in the WDF group (Figure 5E). No changes were observed over time for all groups when LA was normalized by AO (Figure 6F). Overall, most of the observed changed reflect the growth of animals over time, with no significant effect of obesogenic diets on the cardiac structure.

**Figure 7:**
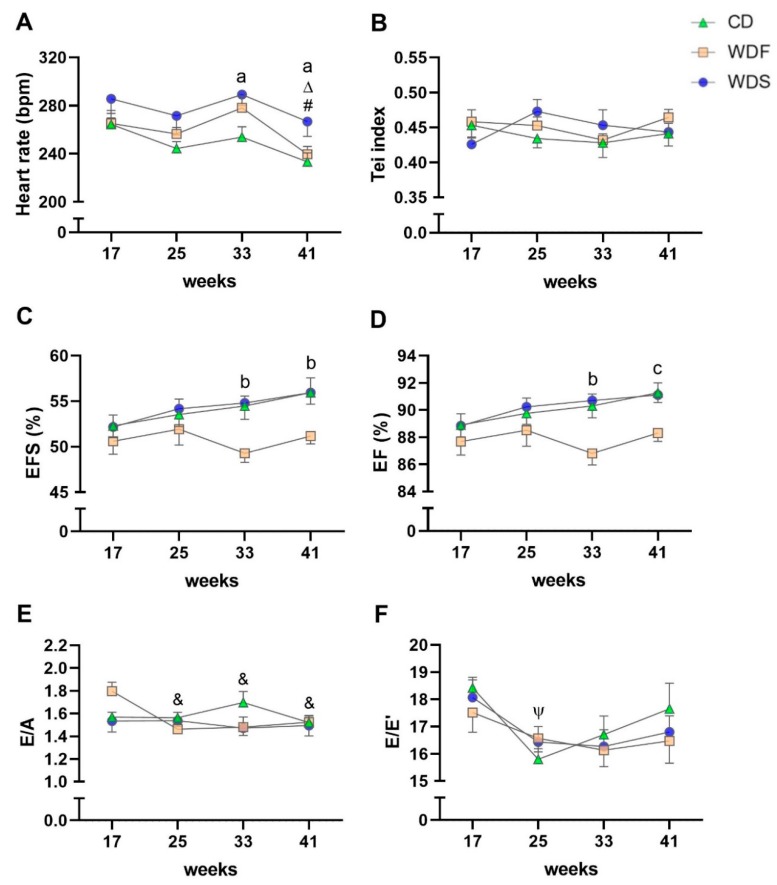
Serial echocardiographic functional assessment. The analysis was performed at 17th, 25th, 33rd, and 41st week of dietary intervention with Control diet (CD), Western diet fat (WDF), and Western diet sugar (WDS). (**A**) Heart rate. (**B**) Tei index. (**C**) Endocardial fractional shortening (EFS). (**D**) Ejection fraction (EF). (**E**) early (E) and late (A) diastolic mitral inflow velocities ratio. (**F**) E and tissue Doppler imaging (TDI) of early mitral annulus diastolic velocity (E’) ratio. Data are presented as mean ± s.e.m. Two-way repeated-measures ANOVA and Bonferroni post hoc test. Symbols indicate differences between the moments fixed the group. Letters indicate differences between the groups fixed the moment. **^#^** *p* < 0.001 vs. 17 weeks for C and WDF; **^Δ^** *p* < 0.05 vs. 33 weeks for WDF; **^&^** vs. 17 weeks for WDF; **^Ψ^** vs. 17 weeks for C; **^a^** C vs. WDS, **^b^** C and WDS vs. WDF; and **^c^** C vs. WDF (*n* = 10 per group).

Regarding cardiac function, no statistical differences were detected among the three groups for the Tei index and E/A and E/E’ ratios (Figure 7B,E,F). HR was higher at the 33rd and 41st weeks in the WDS group compared to CD (Figure 7A). EFS was lower at weeks 33 and 41 in the WDF group in comparison to CD and WDS (Figure 7C). EF also decreased at the 33rd week in the WDF group compared to CD and WDS; however, this variable was reduced more at week 41 in the WDF group than in CD, showing a trend toward declined values concerning WDS (*p* = 0.059) (Figure 7D). In the comparisons among the moments, the HR was lower in the 41st week compared to the 17th and 33rd week in the WDF group; this variable decreased over time in the CD group, being different between the weeks 17 and 41 (Figure 7A). The E/A ratio was reduced at weeks 25, 33, and 41 concerning week 17 in the WDF group (Figure 7E). The E/E ratio decreased significantly from the 17th week to 25th in the CD group (Figure 7F). There was no difference among the moments for the Tei index, EFS, and EF in the three groups (Figure 7B–D).

### 3.4. Cardiac Morphological Evaluation

Post-mortem cardiac macroscopic structure data for the three groups are presented in Table 3. The WDF group showed increased RVW/T and a trend toward increased HW/T (*p* = 0.067) in relation to the CD.

**Figure 8:**
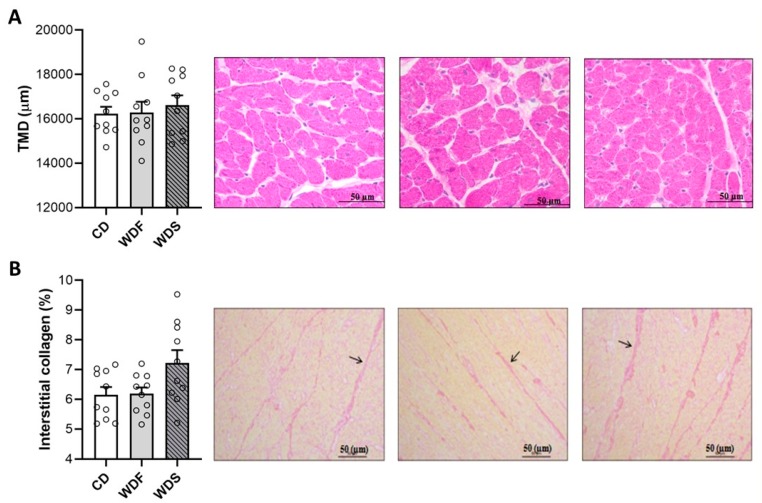
Left ventricle histologic analysis at the 41st week of dietary intervention with Control diet (CD), Western diet fat (WDF), and Western diet sugar (WDS). (**A**) Transverse myocyte diameter (TMD). (**B**) Collagen interstitial fraction (evidenced with arrows). Data are presented as mean ± s.e.m. One-way ANOVA with Tukey post-hoc test (*n* = 10 rats per group).

Regarding the LV histological analysis (Figure 8), our results showed that the rats receiving WDF and WDS had no change in the transverse myocyte diameter and interstitial collagen fraction. These results indicate that the obese rats did not develop cardiac hypertrophy and interstitial collagen accumulation. Of note, there was a tendency to higher values of interstitial collagen fraction in the WDS group compared to the CD (*p* = 0.059).

## 4. Discussion

Obesity is a metabolic disease associated with several comorbidities, including CVD, and its epidemic has reached alarming levels worldwide. Thus, pre-clinically, experimental models of DIO have been widely studied. One of the most commonly used diets is the WD, which may change in the proportion of fat and sugar, as well as in the time-course of obesity and CVD induction, making it challenging to find the most appropriate model. Therefore, in the present study we have directly assessed the effects of two types of WD, one with a higher quantity of fat (WDF) and another higher in sugar (WDS), on cardiac remodeling in obese rats during a long-term follow-up. The main findings of our study were that although both WD (WDF and WDS) caused pronounced obesity and hypertension, only the WDF was able to induce mild cardiac systolic dysfunction after 33 and 41 weeks of dietary treatment without any sign of cardiac hypertrophy.

As expected, both WD substantially triggered obesity in animals after 41 weeks of the dietary treatment since these animals showed increased body weight and WATs compared to control animals. Although measures related to body fat were only obtained at the end of the study, differences in body weight were early observed from the 12th week for WDS group and from the 15th week for WDF. It has been previously showed that body weight changes reflect body fat in rodents [24]. Despite similar energy intake among the three groups studied, the animals fed with WDS and WDF became obese, suggesting that the nutritional quality of the diet is more related to obesity induction than the calories per se. Similarly, Bortolin et al. [9] showed that WD-fed rats gained more weight and adipose tissue than rats fed a control diet, even though the WD group consumed calories resembling a control group, which proposes that weight gain occurred regardless of calorie consumption. This fact could be explained by the fact that saturated fatty acids are usually less oxidized than unsaturated ones, and the nutrients present different thermogenesis, thus favoring fat deposition [25,26,27]. Taken together, our findings evidence that both Western diets, i.e., regardless of the proportion of fat and carbohydrate, were effective in promoting excessive adipose tissue accumulation, which characterizes obesity, in consensus with other studies [9,12,16,28].

Excess adipose tissue is a major contributor to hypertension [29]. In agreement, the results showed that our DIO models increased systolic blood pressure regardless of the higher predominance of fat or sugar in the diet. Several mechanisms are involved in the pathogenesis of obesity-induced hypertension, including adipokine release, insulin resistance, and stimulation of sympathetic nervous system and renin-angiotensin system [30,31]. However, the renin-angiotensin system overactivity has been described as a crucial factor since excessive and dysfunctional adipose tissue leads to increased release of angiotensin II in circulation [32].

Our main goal was to evaluate the effects of two types of WD on cardiac function in rats during a follow-up of 41 weeks to establish an appropriate model for future studies. Surprisingly, our data revealed that only the WDF was able to induce a mild systolic cardiac dysfunction at the 33rd and 41st week, presenting lower values of EFS and EF, and without any change in structural parameters or presence of hypertrophy or fibrosis. Our present findings did not support the expectation that WD would result in pronounced impairment of diastolic and systolic functions over the 41-week experimental period. The cardiac performance in vivo, evaluated by echocardiogram, may be influenced by many factors, such as heart rate, contractility, preload, and afterload [33]. Thus, a possible explanation for the decreased systolic function observed in WDF-fed rats could be related to elevated afterload since these rats presented hypertension. However, the WDS-fed animals also showed increased SBP and yet no functional change in the heart was observed. Therefore, we believe that the alterations observed in the WDF group str related to contractile muscle properties due to excessive fatty acid supply in this diet. Indeed, lipid overload in the heart is associated with cardiac dysfunction by several mechanisms due to lipotoxicity, including alterations in energy metabolism, especially fatty acid β-oxidation, de novo ceramide synthesis, oxidative stress, inflammation, endoplasmic reticulum stress, among others [34].

The idea that a WD is harmful to cardiac function is based on a growing body of evidence. However, studies have also presented controversial dysfunction pattern, because of different variables, such as animal models, dietary composition, cardiac function analysis methodology, and experimental duration. These points should be taken into consideration for the divergent findings when comparing them. The majority of studies that used WD with higher fat predominance, regardless of the experiment duration, demonstrated an important functional impairment of the heart, assessed by isolated heart preparation, hemodynamic evaluation, isolated cardiomyocyte measurements, and cardiac magnetic resonance imaging [11,12,16,35,36]. However, there was no functional alteration when the analysis was performed by echocardiogram [37,38]. These findings reveal the importance of choosing the method in the cardiac functional outcome. Among the studies that employed WD with high carbohydrate content, authors did not show cardiac dysfunction by echocardiographic or hemodynamic evaluation, independently of experimental protocol duration [39,40,41]. Conversely, authors evidenced the presence of relevant systolic and diastolic dysfunction, evaluated by echocardiogram, when the WD high in carbohydrate was associated with 25% fructose or sucrose in drinking water, suggesting a crucial role of sugars [14,42,43,44,45]. Indeed, elevated sugar intake has been associated with greater risks of developing cardiovascular disease [46]. However, experimental studies with high sugar added in the diet [38,47,48] or in the drinking water [49,50,51] have also shown conflicting results regarding the cardiac dysfunction due to the heterogeneity of the experimental protocol. Future investigations should be of reasonable duration, use defined animal models, and improve comparisons concerning results of relevant doses of nutrients on specific outcomes to better understand the effect of sugar consumption in the absence of potential confounding factors. We believe that the synergy between sugar added in drinking water and the Western diet has a more relevant effect on cardiac remodeling in obesity.

Of note, investigations also showed that WD with a balance between fat and carbohydrate and without sugar in drinking water impairs both systolic and diastolic function [13] or causes slight cardiac systolic dysfunction when assessed by echocardiogram. These discrepant outcomes with other authors cited above may be due to protocol duration, animal model or amount and source of fat and sugar.

Regarding the above remarks, it is noteworthy that the lack of cardiac dysfunction at the whole heart level does not necessarily imply a lack of subtle alterations in cardiomyocyte function. Perhaps a functional analysis at the level of isolated heart, myocyte preparations, or papillary muscle could detect cardiac dysfunction that was not observed in our study. The ex vivo functional evaluations could minimize neurohumoral influences that can compensate for changes in heart performance. Dietary modifications, such as introducing sugar into drinking water, could also modify the outcomes found. Therefore, further studies are needed to search for an appropriate DIO model with cardiac dysfunction, widely exploring different techniques of functional analysis of the heart, animal models, diets, and exposure time.

In conclusion, both WD used in the current study (WDF and WDS) were effective in triggering obesity in animals characterized by the high body weight and adiposity; however, only the WDF induced mild cardiac systolic dysfunction after long-term exposure to diet.

## Figures and Tables

**Table 1:** Ingredients of Control diet (CD), Western diet fat (WDF), and Western diet sugar (WDS).

Ingredients (g/kg)	CD	WDF	WDS
Soybean bran	335	344	340
Soybean hull	189	117	117
Corn bran	278	80	80
Dextrin	147	20	20
Fructose	--	100	180
Sucrose	--	50	80
Soybean oil	14	--	--
Palm oil	--	40	30
Palm kernel oil	9	80	49
Lard	--	140	75
Salt	4	8	8
Vitamin and mineral premix	25	25	25

**Table 2:** Nutritional profile of the animals.

Variables	CD (*n* = 10)	WDF (*n* = 10)	WDS (*n* = 10)
Energy intake, kcal/day	92.1 ± 2.7	85.2 ± 2.2	86.8 ± 2.6
Feed efficiency, %	1.39 ± 0.03	1.88 ± 0.05 ***	1.85 ± 0.07 ***
Initial body weight, g	179 ± 8	183 ± 7	186 ± 8
Final body weight, g	544 ± 10	643 ± 20 **	649 ± 24 **
Total body weight gain, g	366 ± 6	460 ± 17 **	463 ± 26 **
Epididymal fat, g	11.1 ± 0.9	19.3 ± 1.9 *	21.5 ± 2.8 **
Retroperitoneal fat, g	12.1 (8.8–16.3)	33.2 (18.9–78.9) **	34.7 (6.5–63.1) **
Visceral fat, g	8.5 ± 0.6	17.1 ± 1.8 **	15.0 ± 1.9 *
WATs, g	32.2 ± 2.2	73.6 ± 9.0 **	72.0 ± 9.3 **
Adiposity index, %	5.9 ± 0.4	11.2 ± 1.0 ***	10.8 ± 1.1 **

Data are presented as mean ± s.e.m. (One-way ANOVA with Tukey *post-hoc* test) or median (Min–Max) (Kruskal-Wallis followed by Dunn’s *post-hoc* test). * *p* < 0.05, ** *p* < 0.01, and *** *p* < 0.001 vs. Control. Abbreviations: CD: control diet; WDF: Western diet fat; WDS: Western diet sugar; WATs: white adipose tissues.

**Table 3:** Macroscopic cardiac remodeling.

Variables	CD (*n* = 10)	WDF (*n* = 10)	WDS (*n* = 10)
Tibia, cm	4.49 ± 0.02	4.54 ± 0.04	4.53 ± 0.06
HW/T, mg/cm	263 ± 5	285 ± 8	271 ± 6
ATW/T, mg/cm	24.7 ± 1.1	25.2 ± 1.1	24.0 ± 0.8
LVW/T, mg/cm	193 ± 5	202 ± 5	195 ± 4
RVW/T, mg/cm	48.7 ± 1.1	57.8 ± 3.3 *	52.4 ± 2.0

Data are presented as mean ± s.e.m. (one-way ANOVA with Tukey *post-hoc* test). * *p* < 0.05 vs. CD. Abbreviations: CD: control diet; WDF: Western diet fat; WDS: Western diet sugar; HW, ATW, LVW, and RVW: heart, atria, left, and right ventricles weights, respectively.

## References

[1] NCD Risk Factor Collaboration (2016). Trends in adult body-mass index in 200 countries from 1975 to 2014: A pooled analysis of 1698 population-based measurement studies with 19·2 million participants. Lancet.

[2] World Health Organization Fact Sheets: Obesity and Overweight. http://www.who.int/news-room/fact-sheets/detail/obesity-and-overweight.

[3] Swinburn B.A., Sacks G., Hall K.D., McPherson K., Finegood D.T., Moodie M.L., Gortmaker S.L. (2011). The global obesity pandemic: Shaped by global drivers and local environments. Lancet.

[4] Varlamov O. (2017). Western-style diet, sex steroids and metabolism. Biochim. Biophys. Acta Mol. Basis Dis..

[5] Upadhyay J., Farr O., Perakakis N., Ghaly W., Mantzoros C. (2018). Obesity as a Disease. Med. Clin. N. Am..

[6] Alpert M.A., Karthikeyan K., Abdullah O., Ghadban R. (2018). Obesity and Cardiac Remodeling in Adults: Mechanisms and Clinical Implications. Prog. Cardiovasc. Dis..

[7] Abel E.D., Litwin S.E., Sweeney G. (2008). Cardiac remodeling in obesity. Physiol. Rev..

[8] Nilsson C., Raun K., Yan F., Larsen M.O., Tang-Christensen M. (2012). Laboratory animals as surrogate models of human obesity. Acta Pharmacol. Sin..

[9] Bortolin R.C., Vargas A.R., Gasparotto J., Chaves P.R., Schnorr C.E., Martinello K.B., Silveira A.K., Rabelo T.K., Gelain D.P., Moreira J.C.F. (2018). A new animal diet based on human Western diet is a robust diet-induced obesity model: Comparison to high-fat and cafeteria diets in term of metabolic and gut microbiota disruption. Int. J. Obes..

[10] Reuter T.Y. (2007). Diet-induced models for obesity and type 2 diabetes. Drug Discov. Today Dis. Model..

[11] Gonçalves N., Silva A.F., Rodrigues P.G., Correia E., Moura C., Eloy C., Roncon-Albuquerque R., Falcão-Pires I., Leite-Moreira A.F. (2016). Early cardiac changes induced by a hypercaloric Western-type diet in “subclinical” obesity. Am. J. Physiol. Heart Circ. Physiol..

[12] Wilson C.R., Tran M.K., Salazar K.L., Young M.E., Taegtmeyer H. (2007). Western diet, but not high fat diet, causes derangements of fatty acid metabolism and contractile dysfunction in the heart of Wistar rats. Biochem. J..

[13] Carbone S., Mauro A.G., Mezzaroma E., Kraskauskas D., Marchetti C., Buzzetti R., Van Tassell B.W., Abbate A., Toldo S. (2015). A high-sugar and high-fat diet impairs cardiac systolic and diastolic function in mice. Int. J. Cardiol..

[14] Panchal S.K., Poudyal H., Waanders J., Brown L. (2012). Coffee extract attenuates changes in cardiovascular and hepatic structure and function without decreasing obesity in high-carbohydrate, high-fat diet-fed male rats. J. Nutr..

[15] Verboven M., Deluyker D., Ferferieva V., Lambrichts I., Hansen D., Eijnde B.O., Bito V. (2018). Western diet given to healthy rats mimics the human phenotype of diabetic cardiomyopathy. J. Nutr. Biochem..

[16] Akki A., Seymour A.-M.L. (2009). Western diet impairs metabolic remodelling and contractile efficiency in cardiac hypertrophy. Cardiovasc. Res..

[17] National Research Council (2011). Guide for the Care and Use of Laboratory Animals.

[18] Vileigas D.F., Harman V.M., Freire P.P., Marciano C.L.C., Sant’Ana P.G., de Souza S.L.B., Mota G.A.F., da Silva V.L., Campos D.H.S., Padovani C.R. (2019). Landscape of heart proteome changes in a diet-induced obesity model. Sci. Rep..

[19] Vileigas D.F., de Deus A.F., da Silva D.C.T., de Tomasi L.C., de Campos D.H.S., Adorni C.S., de Oliveira S.M., Sant’Ana P.G., Okoshi K., Padovani C.R. (2016). Saturated high-fat diet-induced obesity increases adenylate cyclase of myocardial β-adrenergic system and does not compromise cardiac function. Physiol. Rep..

[20] Song J.-X., Ren H., Gao Y.-F., Lee C.-Y., Li S.-F., Zhang F. (2017). Dietary Capsaicin Improves Glucose Homeostasis and Alters the Gut Microbiota in Obese Diabetic ob/ob Mice. Front. Physiol..

[21] Deus A.F., Vileigas D.F., Silva D.C.T., Tomasi L.C., Campos D.H.S., Okoshi K., Padovani C.R., Cicogna A.C. (2019). Cardiac function and intracellular Ca^2+^ handling proteins are not impaired by high-saturated-fat diet-induced obesity. Braz. J. Med. Biol. Res..

[22] Rosa C.M., Gimenes R., Campos D.H.S., Guirado G.N., Gimenes C., Fernandes A.A.H. (2016). Apocynin influence on oxidative stress and cardiac remodeling of spontaneously hypertensive rats with diabetes mellitus. Cardiovasc. Diabetol..

[23] Lang R.M., Bierig M., Devereux R.B., Flachskampf F.A., Foster E., Pellikka P.A., Picard M.H., Roman M.J., Seward J., Shanewise J.S. (2005). Recommendations for chamber quantification: A report from the American Society of Echocardiography’s Guidelines and Standards Committee and the Chamber Quantification Writing Group, developed in conjunction with the European Association of Echocardiograph. J. Am. Soc. Echocardiogr..

[24] Rogers P., Webb G.P. (1980). Estimation of body fat in normal and obese mice. Br. J. Nutr..

[25] Casas-Agustench P., López-Uriarte P., Bulló M., Ros E., Gómez-Flores A., Salas-Salvadó J. (2009). Acute effects of three high-fat meals with different fat saturations on energy expenditure, substrate oxidation and satiety. Clin. Nutr..

[26] Krishnan S., Cooper J.A. (2014). Effect of dietary fatty acid composition on substrate utilization and body weight maintenance in humans. Eur. J. Nutr..

[27] Jéquier E. (2002). Pathways to obesity. Int. J. Obes. Relat. Metab. Disord..

[28] Neves F.A., Cortez E., Bernardo A.F., Mattos A.B.M., Vieira A.K., Malafaia T.O. (2014). Heart energy metabolism impairment in Western-diet induced obese mice. J. Nutr. Biochem..

[29] Kurukulasuriya L.R., Stas S., Lastra G., Manrique C., Sowers J.R. (2008). Hypertension in obesity. Endocrinol. Metab. Clin. N. Am..

[30] Seravalle G., Grassi G. (2017). Obesity and hypertension. Pharmacol. Res..

[31] Dorresteijn J.A.N., Visseren F.L.J., Spiering W. (2012). Mechanisms linking obesity to hypertension. Obes. Rev..

[32] Schütten M.T.J., Houben A.J.H.M., de Leeuw P.W., Stehouwer C.D.A. (2017). The Link between Adipose Tissue Renin-Angiotensin-Aldosterone System Signaling and Obesity-Associated Hypertension. Physiology.

[33] Bers D.M., Borlaug B.A. (2019). Mechanisms of Cardiac Contraction and Relaxation. Braunwald’s Heart Disease: A Textbook of Cardiovascular Medicine.

[34] Sletten A.C., Peterson L.R., Schaffer J.E. (2018). Manifestations and mechanisms of myocardial lipotoxicity in obesity. J. Intern. Med..

[35] Bostick B., Aroor A.R., Habibi J., Durante W., Ma L., DeMarco V.G. (2017). Daily exercise prevents diastolic dysfunction and oxidative stress in a female mouse model of western diet induced obesity by maintaining cardiac heme oxygenase-1 levels. Metabolism.

[36] Bostick B., Habibi J., Ma L., Aroor A., Rehmer N., Hayden M.R., Sowers J.R. (2014). Dipeptidyl peptidase inhibition prevents diastolic dysfunction and reduces myocardial fibrosis in a Mouse model of Western diet induced obesity. Metabolism.

[37] Jeckel K.M., Veeramachaneni D.N.R., Chicco A.J., Chapman P.L., Mulligan C.M., Hegarty J.R. (2012). Docosahexaenoic acid supplementation does not improve Western diet-induced cardiomyopathy in rats. PLoS ONE.

[38] Hecker P.A., Mapanga R.F., Kimar C.P., Ribeiro R.F., Brown B.H., O’Connell K.A., Cox J.W., Shekar K.C., Asemu G., Essop M.F. (2012). Effects of glucose-6-phosphate dehydrogenase deficiency on the metabolic and cardiac responses to obesogenic or high-fructose diets. Am. J. Physiol. Endocrinol. Metab..

[39] Nguyen S., Shao D., Tomasi L.C., Braun A., de Mattos A.B.M., Choi Y.S., Villet O., Roe N., Halterman C.R., Tian R. (2017). The effects of fatty acid composition on cardiac hypertrophy and function in mouse models of diet-induced obesity. J. Nutr. Biochem..

[40] Medford H.M., Chatham J.C., Marsh S.A. (2012). Chronic ingestion of a Western diet increases O-linked-β-N-acetylglucosamine (O-GlcNAc) protein modification in the rat heart. Life Sci..

[41] Marsh S.A., Dell′Italia L.J., Chatham J.C. (2009). Interaction of diet and diabetes on cardiovascular function in rats. Am. J. Physiol. Circ. Physiol..

[42] Qin L., Zhao Y., Zhang B., Li Y. (2018). Amentoflavone improves cardiovascular dysfunction and metabolic abnormalities in high fructose and fat diet-fed rats. Food Funct..

[43] Poudyal H., Campbell F., Brown L. (2010). Olive leaf extract attenuates cardiac, hepatic, and metabolic changes in high carbohydrate-, high fat-fed rats. J. Nutr..

[44] Ferron A., Francisqueti F., Minatel I., Silva C., Bazan S., Kitawara K., Garcia J.L., Corrêa C.R., Moreto F., Ferreira A.L.A. (2018). Association between Cardiac Remodeling and Metabolic Alteration in an Experimental Model of Obesity Induced by Western Diet. Nutrients.

[45] Iyer A., Brown L. (2011). Fermented wheat germ extract (avemar) in the treatment of cardiac remodeling and metabolic symptoms in rats. Evid. Based Complement. Alternat. Med..

[46] Mirtschink P., Jang C., Arany Z., Krek W. (2018). Fructose metabolism, cardiometabolic risk, and the epidemic of coronary artery disease. Eur. Heart J..

[47] Bouchard-Thomassin A.-A., Lachance D., Drolet M.-C., Couet J., Arsenault M. (2011). A high-fructose diet worsens eccentric left ventricular hypertrophy in experimental volume overload. Am. J. Physiol. Heart Circ. Physiol..

[48] Liu L., Huang X., Gao J., Guo Y., Di Y., Sun S. (2018). Improved endogenous epoxyeicosatrienoic acid production mends heart function via increased PGC 1α-mitochondrial functions in metabolic syndrome. J. Pharmacol. Sci..

[49] Lian Y.-G., Zhao H.-Y., Wang S.-J., Xu Q.-L., Xia X.-J. (2017). NLRP4 is an essential negative regulator of fructose-induced cardiac injury in vitro and in vivo. Biomed. Pharmacother..

[50] Wu X., Pan B., Wang Y., Liu L., Huang X., Tian J. (2018). The protective role of low-concentration alcohol in high-fructose induced adverse cardiovascular events in mice. Biochem. Biophys. Res. Commun..

[51] Farah D., Nunes J., Sartori M., Dias D.D., Sirvente R., Silva M.B., Fiorino P., Morris M., Llesuy S., Farah V. (2016). Exercise Training Prevents Cardiovascular Derangements Induced by Fructose Overload in Developing Rats. PLoS ONE.

